# Closure of a Tracheocutaneous Fistula With a Local Turnover Flap Combined With Pregrafted Palatal Mucosa: A Case Report

**Published:** 2016-11-21

**Authors:** Takeshi Kitazawa, Masato Shiba

**Affiliations:** Department of Plastic and Reconstructive Surgery, Matsunami General Hospital, Gifu, Japan

**Keywords:** tracheocutaneous fistula, closure, flap, mucosal graft, staged surgery

## Abstract

**Objectives:** A method of closing a large tracheocutaneous fistula by a combination of a palatal mucosal graft with a turnover adiposal flap is presented. **Methods:** Mucosa of the same size as the tracheal defect was harvested from the hard palate and grafted just caudal to the fistula. After the mucosal graft had taken, a local flap containing the mucosal graft was turned over the tracheal defect facing the mucosa inward of the tracheal lumen. The defect caused by harvesting the flap was closed horizontally. **Results:** The fistula was closed successfully, and 1 year after the operation, the patient had no airway compromise and the operative scar was inconspicuous. **Conclusions:** Although the described method is a 2-stage procedure, it can be used to simply and reliably reconstruct the mucosal layer of the tracheal lumen and overlying skin.

Prolonged endotracheal cannulation sometimes causes a tracheocutaneous fistula that must be closed surgically. A defect of the tracheal lumen should be replaced by the mucous membrane. A surgical procedure for closing a tracheocutaneous fistula using a local flap and palatal mucosal graft as a substitute for endotracheal lining is described. Although this is a 2-stage method, each procedure is simple and reliable. We believe the present method is feasible even for elderly or debilitated patients.

## CASE REPORT

**Figure 1:**
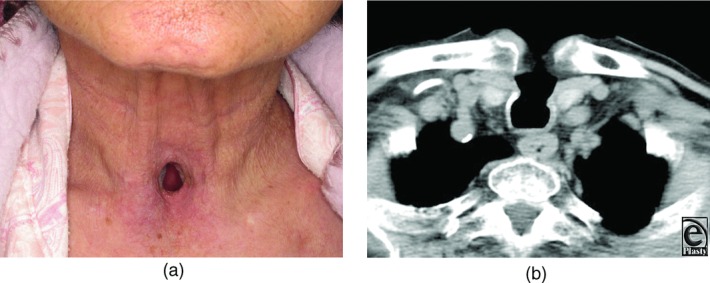
Tracheocutaneous fistula. (a) Preoperative photograph. (b) Computed tomographic scan showing a tracheocutaneous fistula of 15 × 20 mm.

A 78-year-old woman who had a subarachnoid hemorrhage 2 years earlier was referred to our department for closure of a tracheocutaneous fistula. She had undergone tracheostomy to support her breathing with a respirator. At the time she was referred, she had regained clear consciousness and could speak by putting her hand over the fistula. Prolonged intubation left a tracheal wall defect measuring 15 × 20 mm just above the sternal notch (Fig 1*a*). She was constantly coughing up sputum and required tissue paper all day long. Since preoperative computed tomography showed no stenosis or obstruction of the trachea, we decided to simply cover the defect without carrying out a procedure to enlarge the trachea (Fig 1*b*), and resurfacing the inner lumen of the trachea with mucosa to facilitate her expectorating was planned.

## OPERATIVE PROCEDURE

### Stage 1

**Figure 2:**
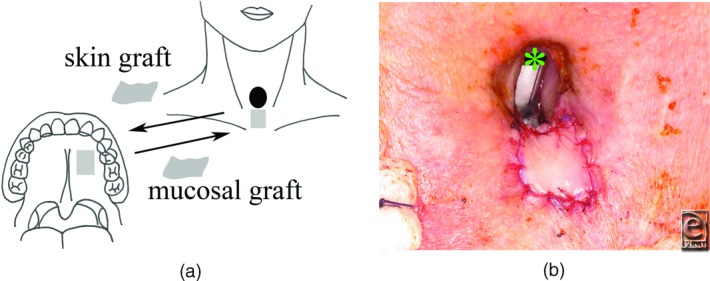
First operation. (a) Schematic drawing of the first operation. Palatal mucosa was grafted just below the orifice of the fistula. Resected skin from the recipient site of the mucosal graft was grafted on the donor site of the palatal mucosa. Gray rectangles indicate skin and mucosal grafts. (b) Mucosal graft measuring 15 × 20 mm. ✽ indicates endotracheal tube.

Skin just below the stoma measuring 15 × 20 mm was removed, and full-thickness mucosa from the hard palate of the same size was grafted on the raw surface following the skin resection. The resected skin was grafted on the donor site of the hard palate. Tie-over gauze was fixed on both grafts (Fig 2).

### Stage 2

**Figure 3:**
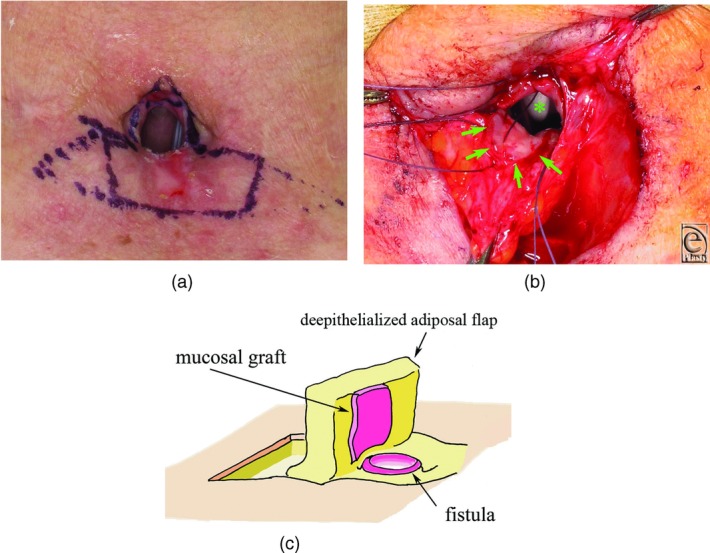
Second operation. (a) Mucosal graft and flap design (solid line) at the second operation. Skin adjoining the mucosal graft is de-epithelialized. Triangular skin outside of the flap (dotted line) is resected to close the wound to a straight line. (b) De-epithelialized flap carrying the mucosal graft (green arrows) is turned over and sutured to the peristomal hinge flap facing the mucosal graft inward. ✽ indicates endotracheal tube. (c) Schematic drawing of the flap.

After it was clear that the mucosal graft had taken, the second operation was performed 17 days after the first operation. A semicircular incision was made around the stoma with a margin of 3 mm and hinged. A rectangular flap measuring 45 × 20 mm containing the mucous graft in the middle of the flap was designed, and the skin beside the pregrafted mucosa was de-epithelialized (Fig 3). The flap was then turned over cephalad, and pregrafted mucosa was sutured to a peristomal hinge to close the fistula with 4-0 absorbable braided polyglactin. The de-epithelialized portion of the flap on both sides was used to fill up the hollow made by the bilateral sternocleidomastoid and sternohyoid muscles. After trimming of the redundant skin and installation of a suction drain, neck skin was sutured horizontally in line with the relaxed tension lines. These 2 operations were performed under general anesthesia with oral intubation.

## RESULTS

**Figure 4:**
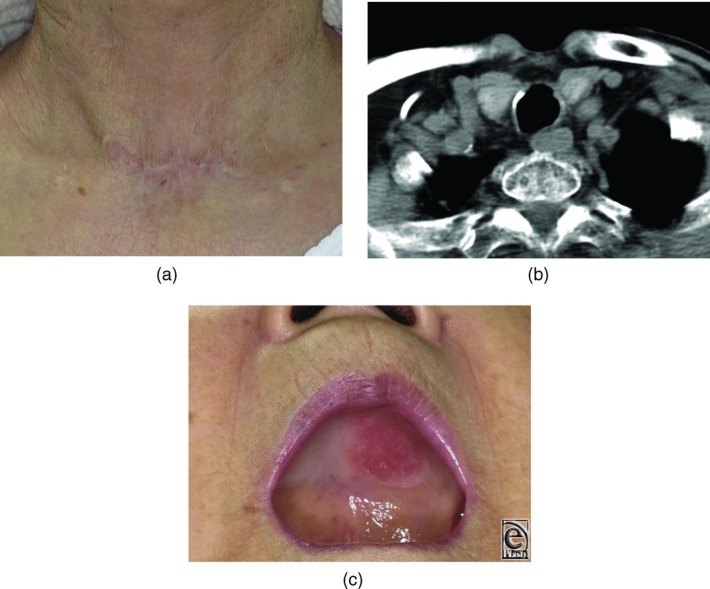
Findings 1 year after surgery. (a) The neck scar is inconspicuous. (b) Computed tomographic scan showing complete closure of the tracheocutaneous fistula without stenosis. (c) Skin graft on the palate is durable, and a full denture could be worn as before.

It took about 1 hour to carry out each operation. The fistula was closed successfully without complications associated with tracheal air or secretion leakage. The skin graft on the hard palate where the mucosal graft had been harvested had taken. Her frequent cough stopped postoperatively. Twelve months after surgery, the patient refused bronchoscopy, but computed tomographic scan showed successful reconstruction. Her neck scar was inconspicuous, and she was very satisfied with the result (Fig 4).

## DISCUSSION

Various methods have been described for closure of tracheocutaneous fistulae. Since simple closure and end-to-end anastomosis have the risks of tracheocele, pneumopericardium, pneumothorax, and pneumomediastinum, their use is limited to patients who have a small defect with good local and general conditions.^1,2^

A peristomal hinged turnover flap to make the inner lining of the trachea with another local flap to cover the skin defect has been reported as a useful method.^3,4^ However, the circumference of the stoma is scar tissue, with poor blood circulation, so the length of the peristomal hinged flap should be limited to a few millimeters. Therefore, this technique was not used in the present case.

Considering the importance of the moisturizing the airway and expectoration, Belsey^5^ stated that a defect of the inner lumen of the trachea is ideally resurfaced with ciliated mucous membrane. However, he had concerns regarding the reconstruction of cylindrical tracheal defects of a certain length, and it remains unclear that the principle should be applied to reconstruction of a tracheocutaneous fistula, that is, a partial defect of the trachea. Some authors have reported the procedure of closure in a tracheocutaneous fistula using mucosa with a flap.^6-8^ Shinohara et al^6^ treated a patient complaining of postoperative difficulty of expectorating who had undergone tracheal reconstruction with a deltopectoral fasciocutaneous flap. The inner layer of the trachea of the patient had been reconstructed with the cutaneous surface of the flap. Since they thought that dirt or sputum on the inner skin layer of the trachea might disturb the respiratory tract, they reconstructed a tracheal defect of another patient with a deltopectoral flap in which the skin island had been replaced by previously grafted palatal mucosa, and the patient had a favorable course without any complications. The watershed of the size of the defect that demands mucosal lining for successful tracheal reconstruction was unknown, but because the present patient had expectorated much sputum preoperatively, a mucosal graft together with a flap was performed. Of course, palatal mucosa, which consists of stratified squamous epithelium and underlying connective tissue, is histologically different from tracheal mucosa, which consists of ciliated epithelium and goblet cells, but it was thought that palatal mucosa could be a suitable substitute for the inner layer of the tracheal lumen.

Yoshimura and Nakajima^7^ reconstructed a tracheal defect using a palatal mucoperiosteal graft. They performed a mucosal graft and overlying neck skin closure simultaneously. They used bolster suture fixation on the skin flap over the mucosal graft with a cotton ball to ensure the taking of the graft. Although their case made good progress after the operation, in the present case, there was concern that this maneuver could jeopardize the blood circulation of the skin flap with the pressure of the cotton ball, and if the mucosal graft did not take, it might become dirt and obstruct the bronchus. For this reason, the palatal mucosa was grafted prior to the flap surgery, and it was confirmed to have taken. Royer et al^8^ reported reconstruction with a prefabricated forearm flap combined with a buccal mucosa and conchal cartilage graft. The reason they chose the free flap for closure was the patient's history of thyroid lymphoma treated with chemotherapy. Since the present case had no history of radiation therapy, a local flap was used.

Defect of palatal mucosa can be epithelialized spontaneously. However, since we secured a piece of skin as a waste generated by preparation for the mucosal grafting, we utilized it for palatal resurfacing. In view of the fact that the skin graft on the palate took in a week, which might be a shorter period than that of spontaneous healing,^7^ we consider the graft on the palate valid in the present case.

Although the fact that the described procedure consists of 2 stages may be a shortcoming, we believe that it can be easily performed with basic surgical techniques to provide reliable physiologic reconstruction of the trachea.
